# Economic Incentives for COVID‐19 Vaccination Among Employees of a Safety‐Net Health System and Medical Center in Southern California: A Cross‐Sectional Study

**DOI:** 10.1002/hsr2.71454

**Published:** 2025-11-09

**Authors:** Dhruv Khurana, Lauren Garcia, Debbie Freund, Anthony Firek, Nicole M. Gatto

**Affiliations:** ^1^ Department of Health Policy & Management, Fielding School of Public Health University of California Los Angeles California USA; ^2^ Comparative Effectiveness and Clinical Outcomes Research Center Riverside University Health System Moreno Valley California USA; ^3^ School of Community & Global Health Claremont Graduate University Claremont California USA; ^4^ Department of Population & Public Health Sciences, Keck School of Medicine University of Southern California Los Angeles California USA; ^5^ School of Public Health Loma Linda University Loma Linda California USA

**Keywords:** COVID‐19, economically based incentives, financial incentive, healthcare workers, monetary reward, paid time off, raffle, Vaccine Hesitancy, vaccine uptake

## Abstract

**Background and Aims:**

Healthcare workers (HCWs) were pivotal in delivering care during the COVID‐19 pandemic, yet vaccination uptake in the United States was lower than anticipated. This study investigated whether economic incentives, paid time off (PTO), raffle entry, or a direct financial incentive could influence vaccine uptake among HCWs exhibiting greater vaccine hesitancy.

**Methods:**

We conducted a cross‐sectional survey in a large integrated safety‐net health system. Using an adapted Vaccine Hesitancy Scale (VHS), employees were classified as “more” vs “less” hesitant. For each incentive, respondents indicated whether it would influence their vaccination decision. Multivariable logistic regression estimated associations between demographic (age, gender, race/ethnicity, household income, education, marital status) and employment factors (job type, COVID‐19 exposure, pandemic impact on income/employment) and reported influence.

**Results:**

Of 684 respondents with complete hesitancy data, 282 (41.2%) were classified as more hesitant. Within this group, 41.4% reported PTO would influence their decision, 28.5% a financial incentive, and 20.3% a raffle. Employees < 50 years had higher odds of reporting influence for a financial incentive (aOR = 3.52; 95% CI: 1.64–8.23), raffle (aOR = 2.34; 1.01–5.41), and PTO (aOR = 1.89; 1.00–3.62). Nonclinical staff were more likely to report influence from a financial incentive than clinical staff (aOR = 2.16; 1.07–4.46). Those reporting negative pandemic‐related income/employment impact showed a borderline association with financial incentives (aOR = 2.12; 0.99–4.59).

**Conclusion:**

Economic incentives are an effective strategy for improving vaccine uptake among younger HCWs, nonclinical staff, and those negatively affected financially by the pandemic. A targeted application of financial incentives may help address vaccine hesitancy in healthcare settings.

**Abbreviations**
aOR: adjusted odds ratio
CI: confidence interval
HCW: healthcare workers
NP: nurse practitioner
OLS: ordinary least squares
OR: odds ratio
PA: physician assistant
PTO: paid time off
RUHS: Riverside University Health System
SAGE: strategic advisory group of experts on immunization
SE: standard error
VHS: vaccine hesitancy scale
WHO: World Health Organization

## 1 Introduction

During the COVID‐19 pandemic, healthcare workers (HCWs) were noted for their dedication to serving patients infected with the SARS‐CoV‐2 virus despite significant risks to themselves and their families [1]. Fortunately, the risk of infection, hospitalization, and death associated with COVID‐19 disease was reduced and prevented by the rapid development and deployment of safe and effective COVID‐19 vaccinations accompanied by public health measures such as masking and physical distancing [2]. However, vaccine acceptance was not uniform among the general population or HCWs in the United States, and many HCWs delayed vaccination with adverse consequences [3, 4, 5]. The decision to refuse or delay vaccination has been a global concern for decades [6]. Understanding and then discovering solutions to reduce vaccine hesitancy among HCWs is critically important, as their vaccination decisions can significantly impact health resources, public health efforts, and overall vaccine uptake in the broader population [7].

HCWs were prioritized for vaccination during the COVID‐19 pandemic, and vaccine hesitancy among HCWs was at a lower rate compared to the general population [8, 9, 10, 11, 12]. However, despite having priority status, voluntary vaccination uptake among U.S. HCWs was not optimal [3, 4]. Many concerns within the general US population related to vaccination were shared by HCWs, including issues with vaccine safety, efficacy, and questions about vaccine development, as well as mistrust in the state, federal, and public health response to COVID‐19 [3, 4, 5].

Interventions have been developed to reduce COVID‐19 vaccine hesitancy among HCWs globally. Still, current research is divided into the most effective approach [13, 14, 15, 16, 17, 18]. Economically oriented incentives, such as a financial payment or direct reward, can “nudge” decision‐making. Ethical concerns have been cited for utilizing financial incentives [15], which can be viewed as coercive [19, 20].

Prior studies examining financial incentives for vaccination have yielded mixed findings across populations and contexts. In the United States, hypothetical experiments indicated that substantial cash payments (e.g., $600–$1200) could increase vaccination intention among hesitant adults, particularly those undecided rather than firmly opposed [21]. By contrast, a randomized controlled trial among Medicaid enrollees in California found that small financial incentives ($10 or $50) and other behavioral interventions (such as nudges) did not increase vaccination uptake, despite improving intentions [22]. Lottery‐based approaches also appear ineffective: in two U.S. experiments, different lottery structures and framings produced no meaningful change in vaccination intentions, and over 40% of participants reported they would not vaccinate for any lottery reward [23]. A population‐level randomized trial in Germany similarly found no direct effect of offering €40 for vaccination and, importantly, identified significant adverse spillover effects on booster uptake [24]. In some U.S. settings, incentives were even perceived negatively: in Ohio, more participants reported that lottery and cash programs decreased rather than increased their vaccination likelihood [25]. Evidence from Europe highlights further heterogeneity: in Italy, both financial and legal incentives increased booster intentions, though health certificates enabling travel or venue access were stronger motivators than cash [26]. Another German experiment showed that legal freedoms had no effect, and only very large payments (€3,250 or more) significantly increased willingness, raising questions about feasibility and efficiency [27]. In Israel, a national survey conducted immediately after the vaccine rollout similarly found that neither monetary rewards nor the green pass significantly increased the probability of immediate vaccination; accessibility and persuasion strategies were more influential [28]. Evidence from influenza vaccination among healthcare workers also points to the potential of behavioral nudges, with a large RCT showing that social norms, reminders, and default framing increased willingness to vaccinate, though effects varied by job type [29]. Complementary evidence from influenza vaccination among older adults shows that modest monetary incentives (10–20 SGD) significantly increased uptake, but larger payments provided no additional benefit, suggesting diminishing returns and highlighting the importance of cost‐effectiveness [30]. Reviews of the literature reinforce this heterogeneity: one systematic review found that large cash incentives and Ohio's Vax‐a‐Million lottery were linked to higher vaccination rates, while smaller incentives, other lotteries, and messaging had limited or no effect [31]. A more recent narrative review concluded that incentives can be effective, particularly in resource‐constrained settings, but may be ineffective or counterproductive in wealthier countries where mistrust in health systems and political institutions plays a larger role; combining tailored incentives with education and transparency is likely to be more effective J [32].

We previously conducted an anonymous cross‐sectional survey to measure COVID‐19 vaccine acceptance, hesitancy, and refusal among employees at a safety net county medical center and system serving a large, under‐resourced, multi‐ethnic population [33]. We identified characteristics associated with vaccine uptake among health system employees, including HCWs, and determined motivations for vaccination and reasons for vaccine hesitancy and refusal [33]. We then found that motivations varied by sociodemographic factors of health system employees and HCWs, including race and ethnicity, household income, and education, among those who accepted vaccination and those who were hesitant or refused [34]. Given the lack of a robust understanding of the effectiveness of economically based incentives among HCWs, in this study, we investigated whether economically based incentives (financial bonuses, raffles, and paid time‐off) influence COVID‐19 vaccine uptake among employees of a safety‐net health system. Subsequently, we investigated the demographic and employment‐related characteristics associated with accepting each economic incentive among employees exhibiting higher vaccine hesitancy.

This study aims to explore how findings on employee characteristics and incentive responsiveness can be used to optimally target economic incentives, informing vaccine promotion strategies by health administrators and policymakers.

## 2 Methods

### 2.1 Study Population

From 15 March to 26 April 2021, we administered a web‐based anonymous survey to assess COVID‐19 vaccine acceptance among employees of the Riverside University Health System (RUHS), an integrated health network in Riverside County, California, as previously described [33, 34]. Of 2983 Medical Center employees who received a link to the survey, 791 submissions were received for a 27% response rate. After excluding two blank or fictitious records, 789 responses were available. This analysis included employees who responded to the vaccine hesitancy scale (see below).

### 2.2 Measures

#### 2.2.1 Vaccine Hesitancy

Vaccine hesitancy was measured using an adapted [33] version of the validated World Health Organization (WHO) Strategic Advisory Group of Experts on Immunization (SAGE) vaccine hesitancy scale (VHS) [35] for use in adults. The VHS comprises eleven items on which respondents rate their opinions using a Likert scale with the response options “strongly disagree,” “disagree,” “neutral,” “agree,” and “strongly agree.” For three items, a “strongly agree” response indicates a higher level of vaccine hesitancy, while the remaining items are reverse‐coded to ensure a consistent scoring interpretation.

Among the 789 respondents, 105 (13.3%) skipped the VHS portion of the survey, leaving 684 individuals for analysis. There were no differences in demographic factors or employment characteristics between those who provided responses and those whose VHS items were missing.

Overall, the mean total score on the VHS was 21.6 (standard deviation [SD] 7.9) and ranged from 11 to 55, with higher scores indicating greater vaccine hesitancy. We used a cut‐off score of 21 to categorize respondents into two groups: “less vaccine‐hesitant” (total score <= 21) and “more vaccine‐hesitant” (total score > 21). This threshold of 21 was selected as it closely approximates the median value in our sample, thereby providing a balanced categorization of respondents.

#### 2.2.2 Vaccination Status

Vaccination status was collected at baseline with the question, “Have you gotten a vaccine against COVID‐19?” (response options: one dose, two doses, or no). Respondents who had not yet been vaccinated were asked about intent to vaccinate and, if not, whether they would consider vaccination at a later date. For analysis, vaccination status was summarized descriptively and not included as a covariate in the primary incentive models because it plausibly lies on the causal pathway between vaccine hesitancy and willingness to accept incentives. Adjusting for such a mediator could bias effect estimates.

#### 2.2.3 Economically Based Influences of Vaccination

Survey respondents were asked about 17 potential influences of a decision to be vaccinated, including three items related to economically based motivations: “If I were to receive a financial incentive” (financial incentive), “If I were entered in a raffle to win a gift card” (raffle), “If I were given paid time off to get the vaccine” [paid time off (PTO)], which were formulated based on prior studies [36, 37, 38, 39]. Respondents ranked how these reasons would motivate their decision to be vaccinated using a five‐point Likert Scale with response options “definitely would not,” “probably would not,” “not sure,” “probably would,” and “definitely would.” For analyses, responses were collapsed into a binary categorical variable with “probably would” and “definitely would” combined into “would” and other responses into “would not.” We considered “not sure” responses in the “would not” category based on a rationale that indecision was indicative of a lack of motivation rather than a positive inclination towards vaccination.

#### 2.2.4 Demographic and Employment Characteristics

Questions on demographic characteristics for self‐described race and ethnicity (Non‐Hispanic Asian, Non‐Hispanic Black, Hispanic, Non‐Hispanic other, Non‐Hispanic White), annual household income (< $50,000, $50,000–$119,999, $120,000 [US Dollars] or higher), and education (less than a college degree, college degree, higher than college degree) were aligned with standard US Census formats [40]. To simplify analyses, variables were collapsed into dichotomous categories for Non‐Hispanic white versus other racial and ethnic groups, income < $90,000 versus >= $90,000 (US Dollars), and less than a college degree versus college degree or higher. Age categories (18–29, 30–49, 50–64, 65+–years) were re‐grouped into < 50 or 50 years and older. Marital status included single, married, civil union, living with a partner, divorced, separated, or widowed. Current job was reclassified into clinicians [nurse, nursing assistant, medical assistant, doctor, physician assistant (PA), nurse practitioner (NP)] or non‐clinicians (allied health personnel, laboratory, respiratory therapy, radiology personnel, administrative, or non‐direct clinical support/admissions and collections clerk, pharmacist, pharmacy tech).

#### 2.2.5 COVID‐19 Exposure and Impact on Employment and Income

Employees' personal direct or perceived risk of exposure to COVID‐19 weekly was assessed as no direct exposure, minimal, moderate, or high exposure, which we recategorized into low exposure (no direct or minimal exposure) and high exposure (moderate or high exposure). Employees were asked to rate the impact of the pandemic on their employment and income using a Likert Scale with response options “severely decreased,” “decreased,” “no effect,” “improved,” and “don't know.” Responses were recategorized as “decreased” (severely decreased and decreased) and “not decreased” (no effect and improved), whereas “don't know” responses (*n* = 7) were not included in the analysis.

### 2.3 Reliability of Survey

Reliability measures have been reported previously [33, 34]. The vaccine hesitancy scale and the items assessing motivations demonstrated excellent internal consistency (standardized Cronbach's alpha = 0.92 and 0.93, respectively).

### 2.4 Statistical Analysis

#### 2.4.1 Descriptives

Frequencies of demographic characteristics, including age, gender (male, female), race and ethnicity, education, household income, marital status, and employment‐related variables of job type and impact of and exposure to COVID‐19 were summarized by the level of vaccine hesitancy (more *vs.* less hesitant). Among the more vaccine‐hesitant employees, and for each of the three economically related reasons for vaccination (financial incentive, raffle, and PTO), we compared demographic characteristics and employment‐related variables between employees using rescaled categories by whether or not each of these reasons would motivate their decision for vaccination. We tested for differences using chi‐square tests of independence or Fisher's exact tests (when cell sizes were small). Throughout, we defined statistical significance using a two‐sided alpha level of 0.05.

#### 2.4.2 Regression Modeling

For multivariate analyses, we employed logistic regression models to examine associations between each economically based influence (financial incentive, raffle, PTO) and demographic/employment factors for HCWs classified as more hesitant. Variables included in multivariate models were age, household income, education, marital status, race and ethnicity, gender, COVID‐19 exposure, the effect of COVID‐19 on employment/income, and job type. We report adjusted odds ratios (ORs) with 95% confidence intervals (CIs) as measures of effect size and precision. Effect sizes reflect the odds of being motivated by each economic incentive, independent of potential confounders.

We performed no formal correction for multiple comparisons because this was an exploratory analysis to identify potential associations rather than testing a single primary hypothesis. All data were analyzed using R version 4.4.2. Our methodological and reporting approach is consistent with the SAMPL (Statistical Analyses and Methods in the Published Literature) guidelines. All primary outcomes and comparisons (i.e., the relationships between economically based motivations and demographic/employment factors among the more vaccine‐hesitant group) were pre‐specified based on our study objectives. Any additional subgroup explorations or post‐hoc comparisons were exploratory, and we highlighted them within the text of the Results where relevant.

#### 2.4.3 Sensitivity Checks

As a sensitivity analysis, we estimated logistic regression models in the full sample using the continuous Vaccine Hesitancy Scale score as the primary predictor of incentive acceptance, to assess whether results were consistent when hesitancy was modeled across its full range rather than dichotomized at a threshold.

#### 2.4.4 Supplementary Analyses (Appendices)

To improve transparency and probe robustness, we report the raw distribution of responses on whether economically based incentives would motivate COVID‐19 vaccination among RUHS Medical Center employees with higher vaccine hesitancy (VHS > 21), stratified by uncollapsed/original demographic and employment characteristics (Appendix Table 1A).

For robustness and detailed checks, logistic models were re‐estimated with age in its original four‐category form because age is a theoretically and empirically important predictor of vaccine attitudes. To address sparse cells and quasi‐separation under this specification, penalized logistic regression with Firth's correction was applied. All other covariates were kept collapsed, as in the main analysis, to preserve model stability and interpretability (Appendix Table 1B).

As a sensitivity check, we also estimated models treating vaccine hesitancy as the outcome and incentive willingness as the predictors for the entire sample. These analyses were conducted solely for descriptive purposes, as the cross‐sectional design precludes causal inference, and participants were not actually exposed to incentives (Appendix Table 1C).

### 2.5 Ethical Approval and Informed Consent

This study was reviewed and approved by the RUHS Institutional Review Board (Protocol #1733159‐2) and classified as exempt due to the use of deidentified data. It was conducted in accordance with the Declaration of Helsinki. All participants provided informed consent before completing the survey. All responses were anonymized, and no identifying information was collected.

## 3 Results

### 3.1 Descriptives

**Table 1:** Characteristics of RUHS medical center employees (*N* = 684) who participated in the COVID‐19 vaccine survey by level of vaccine hesitancy.

Characteristic	Less hesitant (VHS < = 21)	More hesitant (VHS > 21)	*p*
	*N* (%)	*N* (%)	
Total	401 (100%)	282 (100%)	
Age, years			
18–29	52 (13%)	39 (14%)	0.19
30–49	202 (51%)	154 (55%)	
50–64	130 (33%)	84 (30%)	
65+	16 (4%)	4 (1%)	
Gender			
Female	311 (78%)	237 (84%)	0.036
Male	89 (22%)	41 (15%)	
Nonbinary/third gender	1 (0%)	0 (0%)	
Prefer not to answer	0 (0%)	4 (1%)	
Race and ethnicity			
Non‐Hispanic Asian	54 (13%)	30 (11%)	0.001
Non‐Hispanic Black	31 (8%)	23 (8%)	
Hispanic	123 (31%)	116 (41%)	
Non‐Hispanic Native Hawaiian, Pacific Islander, Native American, Alaskan Native, or Other	10 (2%)	13 (5%)	
Non‐Hispanic White	174 (43%)	78 (28%)	
Prefer not to say	9 (2%)	22 (8%)	
Annual household income, US dollars			
Less than 50,000	41 (11%)	56 (21%)	0.001
50,000–89,000	96 (25%)	64 (24%)	
90,000–120,000	72 (19%)	54 (20%)	
120,000 or above	180 (46%)	95 (35%)	
Education			
Less than BA	125 (31%)	140 (50%)	< 0.001
BA	135 (34%)	86 (31%)	
More than a BA	139 (35%)	55 (20%)	
Marital status			
Single	84 (21%)	77 (28%)	0.027
Married or civil union/living with partner	257 (64%)	176 (63%)	
Divorced/separated/widowed/widower	60 (15%)	26 (9%)	
Job type			
Nurse, nursing assistant, medical assistants	149 (38%)	112 (41%)	< 0.001
Doctor, PA, NP	47 (12%)	8 (3%)	
Allied health personnel; laboratory, respiratory therapy, radiology personnel	62 (16%)	28 (10%)	
Administration or non‐direct clinical support/admissions and collections clerk	114 (29%)	119 (43%)	
Pharmacist, pharm tech	20 (5%)	8 (3%)	
COVID‐19 exposure			
No direct	91 (23%)	67 (24%)	0.002
Minimal	93 (23%)	98 (35%)	
Moderate	117 (29%)	59 (21%)	
High	100 (25%)	54 (19%)	
Impact of COVID‐19 on income/employment			
Severely decreased	17 (4%)	14 (5%)	< 0.001
Decreased	47 (12%)	27 (10%)	
No effect	273 (69%)	218 (79%)	
Increased	54 (14%)	13 (5%)	
Don't know	4 (1%)	3 (1%)	
If I were to receive a financial incentive to get the COVID‐19 vaccine			
Definitely would not	156 (39%)	110 (39%)	< 0.001
Probably would not	48 (12%)	49 (17%)	
Not sure	23 (6%)	42 (15%)	
Probably would	56 (14%)	37 (13%)	
Definitely would	113 (29%)	43 (15%)	
If I were entered in a raffle to win a gift card			
Definitely would not	169 (43%)	133 (48%)	< 0.001
Probably would not	61 (15%)	57 (20%)	
Not sure	32 (8%)	33 (12%)	
Probably would	60 (15%)	36 (13%)	
Definitely would	74 (19%)	21 (8%)	
If I were to receive paid time off to get the vaccine			
Definitely would not	108 (27%)	93 (33%)	< 0.001
Probably would not	35 (9%)	34 (12%)	
Not sure	14 (4%)	37 (13%)	
Probably would	76 (19%)	55 (20%)	
Definitely would	162 (41%)	61 (22%)	

(Table 1) A total of 402 (58.8%) employees were classified as less vaccine‐hesitant, and 282 (41.2%) as more vaccine‐hesitant. Overall, 80.1% of participants were female, with no significant gender differences between hesitancy groups (77.6% vs. 84.0%). Less hesitant employees were more likely to hold a bachelor's degree or higher (68.6% vs. 50.2%), report an annual household income ≥USD 120,000 (46.3% vs. 28.7%), and be clinical staff (50.0% vs. 43.6%). In contrast, more hesitant employees were more likely to identify as Hispanic (41.1%), be employed in Nonclinical roles (56.4%), and report no or minimal COVID‐19 exposure (59.4%).

Distributions of incentive acceptance across the original (uncollapsed) demographic and employment categories are shown in Appendix Table 1A.

Among respondents, 44 (5.8%) reported one dose and 585 (77.5%) two doses, while 126 (16.7%) reported no prior COVID‐19 vaccination (valid *N* = 755 of 789). Among those not yet vaccinated, 14/125 (11.2%) said they would get vaccinated if offered, 63/125 (50.4%) said no, and 48/125 (38.4%) were unsure; within the “no/unsure” subgroup, 23/111 (20.7%) would vaccinate later, 40/111 (36.0%) would not, and 48/111 (43.2%) remained unsure.

### 3.2 Economic Incentives Among Vaccine‐Hesitant Employees

**Table 2:** Summary of whether economically based reasons would motivate the decision to receive the COVID‐19 vaccine among RUHS Medical Center employees with greater vaccine hesitancy by demographic factors and employment characteristics.

Characteristic	“If I were to receive a financial incentive”			“If I were entered in a raffle to win a gift card”			“If I were given paid time off to get the vaccine”		
	Would not	Would	*p*	Would not	Would	*p*	Would not	Would	*p*
	*N* (%)	*N* (%)		*N* (%)	*N* (%)		*N* (%)	*N* (%)	
	201 (100%)	80 (100%)		223 (100%)	57 (100%)		164 (100%)	116 (100%)	
Age									
< 50	125 (62%)	67 (85%)	0.0004	145 (65%)	47 (84%)	0.01	103	88 (77%)	0.02
50 or <	76 (38%)	12 (15%)		78 (35%)	9 (16%)		61	27 (23%)	
Household Income									
< 90K	75 (39%)	44 (56%)	0.01	86 (41%)	32 (57%)	0.03	61 (40%)	58 (51%)	0.05
90K or above	115 (61%)	34 (44%)		125 (59%)	24 (43%)		93 (60%)	55 (49%)	
Education									
< BA	96 (48%)	43 (54%)	0.43	107 (48%)	32 (56%)	0.2	80 (49%)	59 (51%)	0.82
BA or more	104 (52%)	37 (46%)		115 (52%)	25 (44%)		83 (51%)	57 (49%)	
Race and ethnicity									
Nonwhite	59 (33%)	19 (24%)	0.23	67 (33%)	11 (20%)	0.06	50	28 (25%)	0.12
Non‐Hispanic White	122 (67%)	59 (76%)		135 (67%)	45 (80%)		96	84 (75%)	
Marital status									
Single	51 (26%)	26 (33%)	0.06	55 (25%)	21 (38%)	0.08	38 (24%)	39 (34%)	0.001
Married	134 (67%)	41 (53%)		147 (66%)	28 (50%)		115 (71%)	59 (51%)	
Divorced	15 (7%)	11 (14%)		19 (9%)	7 (12%)		9 (5%)	17 (15%)	
Gender									
Female	171 (86%)	65 (82%)	0.45	188 (85%)	48 (86%)	1	136 (84%)	100 (87%)	
Male	27 (14%)	14 (18%)		32 (15%)	8 (14%)		25 (16%)	15 (13%)	0.6
COVID‐19 exposure									
Low/minimal	119 (60%)	46 (58%)	0.68	132 (60%)	33 (58%)	0.75	100 (62%)	65 (57%)	0.38
High	78 (40%)	34 (43%)		87 (40%)	24 (42%)		61 (38%)	50 (43%)	
Job type									
Clinician	93 (47%)	26 (33%)	0.04	97 (45%)	22 (39%)	0.46	69 (43%)	50 (44%)	1
Non‐clinician	103 (53%)	52 (67%)		119 (55%)	35 (61%)		90 (57%)	64 (56%)	
Impact of COVID‐19 on income/employment									
Severely decreased/decreased	22 (11%)	19 (24%)	0.007	29 (13%)	12 (22%)	0.15	21 (13%)	19 (17%)	0.5
No effect/increased	171 (89%)	59 (76%)		187 (87%)	43 (78%)		136 (87%)	94 (83%)	

*Note*: Percentages are calculated using non‐missing responses within each column. Denominators, therefore, vary by characteristic and may not sum to the total *N* reported at the top of each column. Missing values were excluded from percentage calculations and statistical tests. *p*‐values were obtained from Pearson's Chi‐squared test with simulated *p*‐values (based on 2000 replicates).

**Table 3:** Associations between acceptance of economically based incentives (financial bonus, raffle, and paid time off) and demographic/employment characteristics among more vaccine‐hesitant employees (VHS > 21), based on univariate and multivariate logistic regression models.

	Financial incentive		Raffle		PTO	
	Unadjusted	Adjusted	Unadjusted	Adjusted	Unadjusted	Adjusted
Age (Ref: 50 or older)						
< 50 years	3.39 (1.78, 6.96)***	3.52 (1.64, 8.23)**	2.81 (1.36, 6.40)**	2.34 (1.01, 5.41)*	1.93 (1.14, 3.33)*	1.89 (1.0, 3.62)+
Household income (Ref: USD 90 K or above)						
< 90 K	1.98 (1.17, 3.40)*	1.23 (0.61, 2.50)	1.94 (1.07, 3.55)*	1.26 (0.59, 2.70)	1.61 (0.98, 2.63)+	1.33 (0.69, 2.55)+
Education (Ref: < BA)						
BA or more	0.79 (0.47, 1.33)	1.02 (0.53, 1.96)	0.73 (0.40, 1.30)	0.91 (0.45, 1.83)	0.93 (0.58, 1.50)	1.37 (0.75, 2.50)
Race & Ethnicity (Ref: Non‐Whites)						
Non‐Hispanic White	1.50 (0.83, 2.79)	1.32 (0.66, 2.69)	2.03 (1.0w, 4.36)+	1.83 (0.84, 4.00)	1.56 (0.91, 2.72)	1.23 (0.67, 2.29)
Marital status (Ref: Single)						
Married	0.6 (0.33, 1.08)+	0.84 (0.41, 1.75)	0.50 (0.26, 0.95)*	0.73, (0.34, 1.55)	0.50 (0.29, 0.86)*	0.65 (0.33, 1.25)
Divorced	1.44 (0.57, 3.57)	2.38 (0.81, 7.16)	0.96 (0.34, 2.55)	1.48 (0.48, 4.57)	1.84 (0.74, 4.80)	2.04 (0.72, 6.14)
Gender (Ref: Female)						
Male	1.36 (0.66, 2.72)	1.17 (0.49, 2.76)	0.98 (0.40, 2.17)	1.20 (0.47, 3.06)	0.82 (0.40, 1.61)	0.80 (0.35, 1.78)
COVID‐19 exposure (Ref: Low/minimal)						
High	1.13 (0.66, 1.91)	1.82 (0.90, 3.71)+	1.10 (0.61, 1.99)	1.22 (0.59, 2.55)	1.26 (0.77, 2.05)	1.45 (0.77, 2.74)
Job Type (Ref: Clinical)						
Nonclinical	1.81 (1.05, 3.16)*	2.16 (1.07, 4.46)*	1.30 (0.72, 2.38)	1.23 (0.59, 2.56)	0.98 (0.60, 1.60)	1.05 (0.56, 1.96)
Impact of COVID‐19 on income/employment(Ref: No effect/increased)						
Severely decreased/decreased	2.50 (1.26, 4.95)**	2.12 (0.99, 4.59)+	1.80 (0.82, 3.74)	1.56 (0.70, 3.50)	1.31 (0.67, 2.57)	1.21 (0.57, 2.55)
*N*		233		233		232
Likelihood ratio χ² test		29.8, *p* < 0.001		15.7, *p* = 0.11		18.3, *p* = 0.05
AIC		280.35		258.58		322.88

*** *p* < 0.001

** *p* < 0.01

* *p* < 0.05.

^+^ *p* < 0.1

(Tables 2 and 3) Because the primary aim of this study was to identify strategies for targeting incentives among employees with higher vaccine hesitancy, we focus first on this subgroup. Within the hesitant sample (*n* = 282), 41.4% reported that paid time off (PTO) would influence their vaccination decision, followed by 28.5% for a financial incentive and 20.3% for a raffle. Below, we present descriptive contrasts and adjusted associations for each incentive type.

#### 3.2.1 Financial Incentive

Vaccine‐hesitant employees who reported that a financial incentive would influence their decision for COVID‐19 vaccination were more likely to be younger than 50 years (85% vs. 62%), have a household income below USD 90,000 (56% vs. 39%), be employed in nonclinical jobs (67% vs. 53%), and to report that COVID‐19 decreased their income or employment (24% vs. 11%) compared to those who would not be influenced. Other characteristics were similar between the two groups (Table 2).

After adjustment for demographic factors and employment characteristics, respondents younger than 50 were more likely to be influenced by a financial incentive than respondents aged 50 or older (OR = 3.52, 95% CI 1.64, 8.23). Similarly, non‐clinicians were more likely to be influenced than clinicians (OR = 2.16, 95% CI 1.07, 4.46). Employees whose income or employment was negatively impacted by COVID‐19 were more likely to be influenced by a financial incentive than those for whom the pandemic had no effect or improved income or employment (OR = 2.12, 95% CI 0.99, 4.59). HCWs who responded having high exposure to COVID‐19 were also more likely to be influenced by a financial incentive than those with low or minimal exposure (OR = 1.82, 95% CI 0.90, 3.71).

#### 3.2.2 Raffle

Compared to those who would not be motivated, vaccine‐hesitant employees who reported that a raffle would influence their decision for COVID‐19 vaccination were more likely to be younger than 50 years (84% vs. 65.0%) and to have a household income below USD 90,000 (57% vs. 41%).

After adjustment for demographic factors and employment characteristics, respondents aged under 50 years were more likely to be influenced by a raffle than respondents older than 50 (OR = 2.34, 95% CI 1.01, 5.41).

#### 3.2.3 Paid Time Off

Compared with vaccine‐hesitant employees who reported that their decision for COVID‐19 vaccination would not be influenced by receiving PTO, vaccine‐hesitant employees who reported that this economically based motivation would affect them were more likely to be younger than 50 years (76.5% vs. 62.8%) and more likely to be non‐partnered (or single: 34% vs. 24%). They were also more likely to report a household income less than USD 90 K (51% vs. 40%).

After adjustment for demographic factors and employment characteristics, respondents under 50 years were more likely to be influenced by receiving PTO to be vaccinated than older respondents (OR = 1.89, 95% CI 1.0, 3.62). Respondents with household income less than USD 90 K were also more likely to be influenced by a PTO (OR = 1.33, 95% 0.69, 2.55).

### Supplementary Analysis

(Table 1B) In models retaining age in four categories (with all other covariates collapsed for stability), the direction of effects was unchanged. Using Firth penalized logistic regression for the raffle outcome to address quasi‐separation, only job type (nonclinical vs clinical) was significantly associated with accepting a financial bonus (AOR = 2.09, 95% CI 1.06–4.23); no covariates met *p* < 0.05 for raffle (NH White AOR = 1.88, 95% CI 0.90–4.20, *p* < 0.10) or PTO.

### Full‐sample Sensitivity Analyses

**Table 4:** Adjusted odds ratios (95% CI) from multivariate logistic regression models examining associations between demographic and employment characteristics and acceptance of economically based incentives (financial bonus, raffle, and paid time off) in the full employee sample, including the continuous Vaccine Hesitancy Scale as the primary regressor (sensitivity analysis).

Variable	Adjusted odds ratio (95% CI)		
	Financial incentive	Raffle	PTO
VHS (Continuous)	0.95 (0.93–0.97)***	0.95 (0.92–0.97)***	0.95 (0.93–0.97)***
Age (Ref: 50 or older)			
< 50 years	2.33 (1.56–3.51)***	1.97 (1.29–3.04)**	2.53 (1.74–3.72)***
Household income (Ref: USD 90K or above)			
< 90K	1.58 (1.05–2.39)*	1.61 (1.05–2.47)*	1.61 (1.07–2.44)*
Education (Ref: < BA)			
BA or more	1.07 (0.73–1.57)	0.93 (0.62–1.39)	1.18 (0.81–1.73)
Race and ethnicity (Ref: Non‐Whites)			
Non‐Hispanic White	1.08 (0.75–1.56)	1.11 (0.76–1.64)	1.16 (0.82–1.66)
Marital status (Ref: Single)			
Married	1.14 (0.73–1.79)	1.11 (0.70–1.79)	1.01 (0.64–1.57)
Divorced	1.36 (0.73–2.54)	1.24 (0.65–2.36)	1.19 (0.64–2.22)
Gender (Ref: Female)			
Male	1.07 (0.68–1.68)	0.75 (0.45–1.21)	0.80 (0.51–1.26)
COVID‐19 exposure (Ref: Low/minimal)			
High	1.00 (0.66–1.50)	1.02 (0.67–1.57)	1.07 (0.71–1.59)
Job type (Ref: Clinical)			
Nonclinical	1.39 (0.92–2.10)	1.24 (0.81–1.91)	1.15 (0.77–1.73)
Impact of COVID‐19 on income/employment (ref: no effect/increased)			
Severely decreased/decreased	1.39 (0.88–2.20)	1.03 (0.63–1.66)	1.20 (0.75–1.92)
*N*	589	589	587
Likelihood ratio χ² test, *p*‐value	50.09, *p* < 0.001	37.04, *p* < 0.001	57.44, *p* < 0.001
McFadden's R^2^	0.064	0.052	0.071
AIC	756.35	705.37	777.45

^+^ *p* < 0.1.

*** *p* < 0.001

** *p* < 0.01

* *p* < 0.05,

(Table 4 and Appendix Table 1C) To assess robustness, we estimated models on the full employee sample, treating the Vaccine Hesitancy Scale (VHS) as a continuous covariate. Across all three incentives, higher VHS scores were consistently associated with lower odds of reporting incentive influence (OR ≈ 0.95 per one‐point increase on the vaccine hesitancy scale, *p* < 0.001). Younger employees and those with lower household income were also more receptive to incentives, consistent with subgroup findings. Other demographic and occupational characteristics showed no consistent associations as reported previously.

As detailed in Table 1C, in models estimated separately for each incentive, respondents who reported willingness to accept a financial incentive, PTO, or raffle entry had significantly lower vaccine hesitancy scores on average than those who indicated they would not accept the respective incentive, controlling for sociodemographic and occupational characteristics. Specifically, willingness to accept a financial incentive was associated with a 2.58‐point lower hesitancy score (*p* < 0.001), PTO with a 2.72‐point lower score (*p* < 0.001), and raffle entry with a 2.66‐point lower score (*p* < 0.001). In the fully adjusted model including all three incentives simultaneously (Model IV), PTO remained significantly associated with reduced hesitancy (–1.75, *p* < 0.05), while the coefficients for financial incentives (–0.67) and raffle entry (–1.17) were attenuated and no longer statistically significant. Respondents with a bachelor's degree or higher reported substantially lower hesitancy across all models (–2.35 to –2.44, *p* < 0.001). In contrast, Non‐Hispanic White respondents reported higher hesitancy compared to non‐Whites (about 1.40, *p* < 0.05). Divorced respondents reported higher hesitancy than single respondents (2.5, *p* < 0.05), as did men compared to women (1.8, *p* < 0.05). Greater COVID‐19 exposure was also associated with higher hesitancy (1.5, *p* < 0.05). Nonclinical staff reported marginally higher hesitancy relative to clinical staff (1.14–1.30, *p* < 0.10). No significant associations were observed for age (< 50 vs. ≥ 50), household income (<$90K vs. ≥$90K), marital status (married vs. single), or the impact of COVID‐19 on income/employment.

## 4 Discussion

Among employees and HCWs at a major integrated safety net health system, we found that paid time off, raffle, and direct payment of a financial incentive had a variable influence on the decision to vaccinate among those who were more hesitant about COVID‐19 vaccination. After adjusting for demographic and employment characteristics, age was consistently associated with all three economically based incentives. Our results suggested that economically based incentives would be most effective if targeted to those who are younger than 50 years of age. Employees with a nonclinical job type were also motivated by a financial incentive, but not by PTO or being entered into a raffle. In the full‐sample models treating hesitancy continuously, higher VHS scores were consistently associated with lower odds of reporting that any incentive would matter, reinforcing that incentives are least compelling for those with stronger baseline hesitancy.

HCWs are the backbone of the healthcare delivery system, and it is imperative to understand the motivations driving vaccine uptake among HCWs to identify specific interventions that may improve vaccine acceptance. Ignoring vaccination can profoundly impact health outcomes, have economic implications, and lead to a general disruption of the social fabric [41, 42]. During the COVID‐19 pandemic, economically based incentives were employed to increase vaccination uptake. This strategy was widely used in the general population, with many US states introducing economic incentive programs ranging from small, guaranteed rewards to lotteries [43]. Results from Ohio's Vax‐A‐Million program showed that raffling USD 1,000,000 and one full‐ride scholarship to any public college or university successfully increased vaccine uptake, especially among persons living in vaccine‐hesitant areas of the state [44, 45, 46]. However, even though the Vax‐a‐Million program resulted in an additional 114,553 Ohioans receiving vaccinations, most remained unvaccinated by the end of the program [46]. Other US states did not detect a significant increase in COVID‐19 vaccination rates when they adopted lottery‐based incentives [47, 48, 49].

Government officials in Detroit, Michigan, offered a USD 50 prepaid card to individuals who received the COVID‐19 vaccine. They found it successful at increasing the weekly vaccination rate by 44.19% for the first dose but ineffective for the second [14]. A pilot program in North Carolina provided a guaranteed USD 25 cash card to adults who either received or drove someone to their first COVID‐19 vaccination dose and reported that incentives supported more equitable distribution, especially among low‐income persons or persons of Black or Hispanic race and ethnicity C. A [50]. Another study reported an 8% increase in willingness to receive the vaccine with cash amounts of USD 1000, USD 1500, or USD 2000. Yet, increases were not relative to the cash incentive amount [51]. Other cross‐sectional surveys conducted in the US found that offering a USD 10 or USD 100 financial incentive would not increase the willingness of individuals to vaccinate against COVID‐19 [52]. At the same time, payments of USD 500 or more would be necessary [53]. As data have been inconsistent on the effectiveness of various economic‐based incentive mechanisms for the public, uncertainty persists regarding how best to implement such approaches within health system settings.

HCWs are generally recognized as having altruistic motivations, and public health administrators must balance encouraging HCWs' altruism with providing financial incentives to boost overall vaccination rates. In addition, medical systems will need to weigh the benefits against the costs related to resource expenditures in offering additional direct monetary payments to employees to encourage vaccination. A study among HCWs in Massachusetts demonstrated that many had negative attitudes toward using financial incentives to increase vaccination rates, viewing them as coercive and potentially undermining public trust in vaccinations [54]. Furthermore, other studies have suggested that providing economic incentives to HCWs may not be a practical approach to increasing vaccination rates. For example, a study in Austria concluded that monetary incentives did not motivate nursing and social care employees, regardless of the amount provided [55]. This highlights a significant challenge in considering the potential effectiveness of financial incentives, ethical concerns, and perceptions of incentivizing HCWs. Despite the debate over incentives to improve personal health decisions within the general population, more information is needed about the acceptance and outcomes of this approach among HCWs.

Our observation that younger HCWs will respond favorably to economic incentives should be investigated thoroughly to understand the underlying drivers of this relationship. A possible explanation could be the pay differences between younger HCWs and older HCWs. One study found that younger HCWs during the COVID‐19 pandemic were more likely to report burnout, with a portion of the burnout stemming from economic hardships [56], which is prevalent among younger HCWs [57]. Providing economic incentives to younger health system employees could help to moderate financial stressors.

Our observation of increased motivation from a cash incentive among non‐clinicians compared to clinicians could relate to the nature and level of stress each role experienced during the COVID‐19 pandemic. Financial strain was correlated with posttraumatic stress symptoms among nonclinical HCWs related to COVID‐19 in one study [58]. Pay gaps could also explain why nonclinical HCWs would be attracted to cash incentives. Other research highlighted that nonclinical health roles were severely underpaid during the COVID‐19 pandemic, with more than 20% living in poverty and 40% relying on public assistance [59].

These results should be interpreted in context. The survey was administered when COVID‐19 vaccines were first introduced in the US; perspectives on economically based incentives could have evolved and would not be reflected in our data. The county health system environment provided strong employment protections, effectively guaranteeing job security, income, and benefits during this period; these factors may have tempered ratings of incentive attractiveness. Because the primary analyses were restricted to health care workers classified as vaccine‐hesitant, most respondents had not completed vaccination at the time of the study. Although the survey phrasing (“If I were to receive…”) was consistent across groups, vaccinated participants, when included (in sensitivity analyses), would have interpreted these items hypothetically. Thus, the results should be understood as reflecting general hypothetical willingness rather than real‐time decision‐making about initial vaccine uptake. Missing data reduced the effective sample size for multivariable models, and sparse cells, especially when retaining the full age categorization, required penalized logistic regression for raffle models; wide confidence intervals around several estimates warrant cautious interpretation. Although vaccination status was measured, we summarized it descriptively due to its close linkage with hesitancy and its potential position on the causal pathway between hesitancy and stated responsiveness to incentives.

Building on our findings, we propose several actionable recommendations for health administrators and policymakers: Because employees under 50 years showed greater responsiveness to economically based incentives, offering direct financial incentives such as cash vouchers or gift cards may be especially effective in this demographic. At the same time, nonclinical staff showed a stronger preference for direct monetary rewards, which suggests that administrators could consider developing separate incentive programs to address these different preferences. Incentives can be paired with broader supportive interventions (e.g., stress management resources). This integrated approach could increase receptiveness among hesitant employees. Policymakers should pilot incentive strategies in controlled settings to measure their cost‐effectiveness and impact on vaccination rates. These evaluations can help ensure that initiatives remain ethically sound and appropriately communicated. By following these recommendations, health systems and government agencies can develop a more transparent framework for implementing incentives tailored to the specific needs and characteristics of their workforce. Future research could incorporate broader measures of nonmedical determinants of health, such as the Whole PERSON Health Score, which assesses needs across financial, social, and behavioral domains. Given that nonclinical administrative staff earning under $90,000 annually may experience greater unmet needs in these areas, they may also demonstrate higher responsiveness to financial or time‐based incentives for vaccination [60]. Future research should investigate how these approaches perform across various healthcare settings and over longer timescales to validate and refine their effectiveness. Lastly, given mixed evidence and cost considerations, systems should pilot programs, track uptake and equity impacts, and evaluate cost‐effectiveness before scaling.

## 5 Conclusion

Economic incentives for vaccination may present an effective strategy to address potential vaccination uptake disparity among younger and nonclinical health system workers, yet underlying motivations should be further understood. With this information, practitioners can shape policies and interventions targeted to HCWs to improve vaccination rates. These discoveries may extend to other public health initiatives, such as increasing flu vaccination rates among health system workers.

## Author Contributions

**Dhruv Khurana:** conceptualization, methodology, software, data curation, investigation, formal analysis, writing – review and editing, supervision. **Lauren Garcia:** writing – original draft. **Debbie Freund:** writing – review and editing. **Anthony Firek:** conceptualization, methodology, investigation, supervision, funding acquisition, project administration, resources, writing – review and editing. **Nicole M Gatto:** investigation, conceptualization, methodology, validation, supervision, project administration, resources, writing – review and editing, funding acquisition.

## Conflicts of Interest

The authors report no financial or personal conflicts of interest relevant to this study. No external parties were involved in any aspect of the study design, data collection, analysis, interpretation, writing of the report, or the decision to submit the article.

## Transparency Statement

The lead author Dhruv Khurana affirms that this manuscript is an honest, accurate, and transparent account of the study being reported; that no important aspects of the study have been omitted; and that any discrepancies from the study as planned (and, if relevant, registered) have been explained.

## Data Availability

The data presented in this study are available on request from the corresponding author.

## Appendix

**Table 1A:** Distribution of responses on whether economically based incentives would motivate COVID‐19 vaccination among RUHS Medical Center employees with greater vaccine hesitancy (VHS > 21), shown by original (uncollapsed) demographic and employment characteristics.

Characteristic	“If I were to receive a financial incentive”			“If I were entered in a raffle to win a gift card”			“If I were given paid time off to get the vaccine”		
	Would not	Would	*p*	Would not	Would	*p*	Would not	Would	*p*
	*N* (%)	*N* (%)		*N* (%)	*N* (%)		*N* (%)	*N* (%)	
Total	201 (100%)	80 (100%)		223 (100%)	57 (100%)		164 (100%)	116 (100%)	
Age, years									
18–29	20 (10.0%)	19 (24%)		22 (9.9%)	17 (30%)		17 (10%)	22 (19%)	
30–49	105 (52%)	48 (61%)		123 (55%)	30 (54%)		86 (52%)	66 (57%)	0.02
50–64	73 (36%)	11 (14%)		74 (33%)	9 (16%)		59 (36%)	25 (22%)	
65+	3 (1.5%)	1 (1.3%)		4 (1.8%)	0 (0%)	< 0.001	2 (1.2%)	2 (1.7%)	
Household income, USD									
Less than 50K	33 (17%)	23 (29%)		38 (18%)	18 (32%)		25 (16%)	31 (27%)	
50K–89K	42 (22%)	21 (27%)		48 (23%)	14 (25%)		36 (23%)	27 (24%)	
90K–120K	45 (24%)	9 (12%)	0.032	44 (21%)	10 (18%)	0.081	32 (21%)	22 (19%)	0.1
120 or above	70 (37%)	25 (32%)		81 (38%)	14 (25%)		61 (40%)	33 (29%)	
Education									
Less than BA	96 (48%)	43 (54%)		107 (48%)	32 (56%)		80 (49%)	59 (51%)	
BA	64 (32%)	22 (28%)	0.7	69 (31%)	16 (28%)	0.5	52 (32%)	34 (29%)	0.9
More than a BA	40 (20%)	15 (19%)		46 (21%)	9 (16%)		31 (19%)	23 (20%)	
Race and ethnicity									
Prefer not to say	0 (0%)	0 (0%)		0 (0%)	0 (0%)		0 (0%)	0 (0%)	
Non‐Hispanic White	20 (10%)	2 (2.7%)		21 (9.7%)	1 (1.9%)		18 (11%)	4 (3.7%)	
NH Asian	59 (30%)	19 (26%)	0.048	67 (31%)	11 (21%)	0.1	50 (31%)	28 (26%)	
NH Black	24 (12%)	6 (8.1%)		24 (11%)	6 (12%)		17 (11%)	13 (12%)	0.1
NH Hispanic	18 (9.2%)	5 (6.8%)		19 (8.8%)	4 (7.7%)		13 (8.2%)	9 (8.3%)	
NH Natives	74 (38%)	42 (57%)		85 (39%)	30 (58%)		61 (38%)	55 (50%)	
Marital status									
Single	51 (26%)	26 (33%)		55 (25%)	21 (38%)		38 (23%)	39 (34%)	
Married/partner	134 (67%)	41 (53%)		147 (67%)	28 (50%)		115 (71%)	59 (51%)	
Divorced/separated/widowed	15 (7.5%)	11 (14%)	0.057	19 (8.6%)	7 (13%)	0.073	9 (5.6%)	17 (15%)	0.002
Gender									
Female	171 (85%)	65 (81%)		188 (84%)	48 (84%)		136 (83%)	100 (86%)	
Male	27 (13%)	14 (18%)		32 (14%)	8 (14%)		25 (15%)	15 (13%)	
Nonbinary/third gender	0 (0%)	0 (0%)	0.8	0 (0%)	0 (0%)	> 0.9	0 (0%)	0 (0%)	0.7
Prefer not to answer	3 (1.5%)	1 (1.3%)		3 (1.3%)	1 (1.8%)		3 (1.8%)	1 (0.9%)	
Exposure									
No direct exposure	45 (23%)	22 (28%)		50 (23%)	17 (30%)		36 (22%)	31 (27%)	
Minimal exposure	74 (38%)	24 (30%)		82 (37%)	16 (28%)		64 (40%)	34 (30%)	
Moderate exposure	41 (21%)	17 (21%)	0.7	48 (22%)	9 (16%)	0.2	34 (21%)	24 (21%)	0.3
High exposure	37 (19%)	17 (21%)		39 (18%)	15 (26%)		27 (17%)	26 (23%)	
Job title									
Nurse/Assistants	85 (43%)	26 (33%)		89 (41%)	22 (39%)		63 (40%)	48 (42%)	
Doctor/PA/NP	8 (4.1%)	0 (0%)		8 (3.7%)	0 (0%)		6 (3.8%)	2 (1.8%)	
Allied Health/Lab/Respiratory/Radiology	19 (9.7%)	9 (12%)	0.14	22 (10%)	6 (11%)	0.5	14 (8.8%)	14 (12%)	0.7
Admin/Nonclinical support	79 (40%)	40 (51%)		92 (43%)	26 (46%)		72 (45%)	46 (40%)	
Pharmacist/Tech	5 (2.6%)	3 (3.8%)		5 (2.3%)	3 (5.3%)		4 (2.5%)	4 (3.5%)	
Other	0 (0%)	0 (0%)		0 (0%)	0 (0%)		0 (0%)	0 (0%)	
Impact of COVID‐19 on income/employment									
Severely decreased	7 (3.6%)	7 (8.8%)		9 (4.1%)	5 (8.8%)		8 (5.1%)	6 (5.2%)	
Decreased	15 (7.7%)	12 (15%)		20 (9.2%)	7 (12%)		13 (8.2%)	13 (11%)	
No effect	163 (84%)	54 (68%)	0.023	179 (82%)	38 (67%)	0.029	130 (82%)	87 (76%)	0.6
Improved	8 (4.1%)	5 (6.3%)		8 (3.7%)	5 (8.8%)		6 (3.8%)	7 (6.1%)	
I don't know	1 (0.5%)	2 (2.5%)		1 (0.5%)	2 (3.5%)		1 (0.6%)	2 (1.7%)	

**Table 1B:** Associations between acceptance of economically based incentives (financial bonus, raffle, and paid time off) and demographic/employment characteristics among more vaccine‐hesitant employees (VHS > 21), based on multivariate logistic regression models with age as the original categorical predictor.

Variable	Adjusted odds ratio (95% CI)		
	Financial incentive	Raffle	PTO
Age (Ref: 65 or older)			
18–29	3.21 (0.42, 39.41)	7.13 (0.62, 996.62)	1.44 (0.18, 11.68)
30–49	1.39 (0.20, 15.87)	2.39 (0.23, 325.39)	0.87 (0.12, 6.43)
50–64	0.48 (0.07, 5.58)	1.41 (0.13, 194.51)	0.50 (0.07, 3.77)
Household income (Ref: USD 90K or above)			
< 90K	1.18 (0.59, 2.36)	1.26 (0.60, 2.64)	1.28 (0.67, 2.42)
Education (Ref: < BA)			
BA or more	1.08 (0.57, 2.09)	1.04 (0.52, 2.11)	1.38 (0.77, 2.50)
Race and ethnicity (Ref: Non‐Whites)			
Non‐Hispanic White	1.41 (0.71, 2.84)	1.88 (0.90, 4.20)+	1.28 (0.70, 2.35)
Marital status (Ref: Single)			
Married	0.98 (0.47, 2.05)	0.92 (0.43, 2.00)	0.71 (0.37, 1.37)
Divorced	2.85 (0.98, 8.44)+	2.12 (0.67, 6.51)	2.15 (0.77, 6.28)
Gender (Ref: Female)			
Male	1.19 (0.50, 2.73)	1.22 (0.47, 2.97)	0.81 (0.36, 1.75)
COVID‐19 exposure (Ref: Low/minimal)			
High	1.73 (0.88, 3.45)	1.19 (0.59, 2.43)	1.41 (0.77, 2.63)
Job Type (Ref: Clinical)			
Nonclinical	2.09 (1.06, 4.23)*	1.24 (0.61, 2.55)	1.05 (0.57, 1.94)
Impact of COVID‐19 on income/employment (ref: no effect/increased)			
Severely decreased/decreased	2.04 (0.97, 4.31)+	1.52 (0.67, 3.31)	1.19 (0.58, 2.45)
*N*	233	233	232
Likelihood ratio χ² test, *p*	32.62, *p* = 0.00	21.41, *p* = 0.04	19.36, *p* = 0.08
Wald Test, *p*	46.29, *p* = 0.00	62.20, *p* = 0.00	19.35, *p* = 0.08

*Note:* **p* < 0.05; ****p* < 0.001; ***p* < 0.01; ^+^ *p *< 0.1.

**Table 1C:** Association between vaccine hesitancy scale scores and willingness to accept financial incentives, paid time off, or raffle to get the COVID‐19 vaccine among healthcare workers, controlling for available demographics and employment characteristics.

Variable	I	II	III	IV
Financial incentive (Ref: would not)				
Would	−2.58 (0.60)***	‐‐‐	‐‐‐	−0.67 (0.87)
PTO (Ref: would not)				
Would	‐‐‐	−2.72 (0.67)***	‐‐‐	−1.75 (0.84)*
Raffle (Ref: would not)				
Would	‐‐‐	‐‐‐	−2.66 (0.59)***	−1.17 (0.80)
Age (Ref: 50 or older)				
< 50 years	−0.27 (0.72)	‐0.36 (0.76)	−0.15 (0.72)	−0.41 (0.75)
Household Income (Ref: USD 90K or above)				
< 90K	−0.59 (0.72)	−0.63 (0.72)	−0.59 (0.72)	−0.73 (0.72)
Race and ethnicity (Ref: Non‐Whites)				
Non‐Hispanic White	1.40 (0.67)*	1.39 (0.67)*	1.41 (0.67)*	1.40 (0.67)*
Education (Ref: < BA)				
BA or more	−2.37 (0.66)***	−2.35 (0.65)***	−2.44 (0.66)***	−2.35 (0.65)***
Marital status (Ref: Single)				
Married	−0.06 (0.88)	−0.20 (0.88)	−0.07 (0.88)	−0.14 (0.88)
Divorced	−2.43 (1.06)*	−2.57 (1.07)*	−2.48 (1.06)*	−2.48 (1.07)*
Gender (Ref: Female)				
Male	−1.62 (0.83)*	−1.83 (0.82)*	−1.81 (0.83)*	−1.82 (0.83)*
COVID‐19 exposure (Ref: Low/minimal)				
High	−1.53 (0.66)*	−1.48 (0.66)*	−1.51 (0.66)*	−1.48 (0.66)*
Job type (Ref: Clinical)				
Nonclinical	1.30 (0.69)+	1.14 (0.68)+	1.23 (0.69)+	1.21 (0.68)+
Impact of COVID‐19 on income/employment (ref: no effect/increased)				
Severely decreased/decreased	−0.77 (0.91)	−0.89 (0.90)	−0.95 (0.89)	−0.87 (0.91)
*N*	589	587	589	586
Adjusted R^2^	0.09	0.09	0.09	0.1
F‐statistic	6.418	6.68	6.36	6.002

*Note:* Each column represents a separate regression. Values are coefficients with standard errors in parentheses. OLS with robust (HC1) standard errors. Models adjust for age (4‐category), gender, race/ethnicity, household income, education, marital status, job title, exposure, and perceived income change. VHS is treated as continuous; higher scores indicate greater vaccine hesitancy.

***p* < 0.01

*** *p* < 0.001

* *p* < 0.05.

^+^ *p* < 0.1.
